# Multiple Injuries to the Lower Urinary Tract: Two Cases and Comparison with the EAU Guidelines

**DOI:** 10.1155/2018/3216527

**Published:** 2018-12-18

**Authors:** N. Lumen, D. Sharma, Y. Abu-Ghanem, N. Djakovic, F. Kuehhas, E. Serafetinidis, A. Sujenthiran, M. Waterloos, P. Hallscheidt, N. D. Kitrey

**Affiliations:** ^1^Dept. of Urology, Ghent University Hospital, Ghent, Belgium; ^2^Dept. of Urology, St George's University Hospitals NHS Foundation Trust, London, UK; ^3^Dept. of Urology, Sheba Medical Centre, Tel-Hashomer, Israel; ^4^Dept. of Urology, General Hospital Mühldorf, Mühldorf, Germany; ^5^Fachartz für Urologie und Andrologie, Vienna, Austria; ^6^Dept. of Urology, Asclepeion Hospital, Voula, Greece; ^7^Dept. of Urology, Algemeen Ziekenhuis Maria Middelares, Ghent, Belgium; ^8^Dept. of Radiology, Darmstadt, Germany

## Abstract

Blunt trauma to the lower urinary tract is usually associated with pelvic fractures. The European Association of Urology (EAU) provides guidelines to diagnose and treat these injuries. The guidelines summarise the available evidence and provide recommendations on diagnosis and treatment of these patients. Therefore, these guidelines are important adjuncts to the urologist and emergency physician in the clinical decision-making. However, strict adherence to the guidelines is not always easy or possible because of concomitant injuries obscuring the clinical picture. This is illustrated by two case reports of concomitant injuries of the lower urinary tract (bladder with urethral injury). The clinical decisions will be discussed point by point and should serve as a practical teaching moment for the reader.

## 1. Introduction

Trauma to the lower urinary tract is frequently associated with other injuries [1, 2]. Although lower urinary tract injuries are not directly life-threatening, prompt diagnosis and management are important to prevent further morbidity and even mortality [1, 3]. Two recent cases demonstrate the challenges faced when managing these injuries. The cases are discussed vis a vis the current guidelines of the European Association of Urology (EAU) (http://uroweb.org/guideline/urological-trauma/). The aim of these 2 cases is to provide the reader insights on how these EAU guidelines should be followed in multiple injuries to the lower urogenital tract and to demonstrate where difficulties can be encountered to adhere to these guidelines.

## 2. Case 1

**Figure 1:**
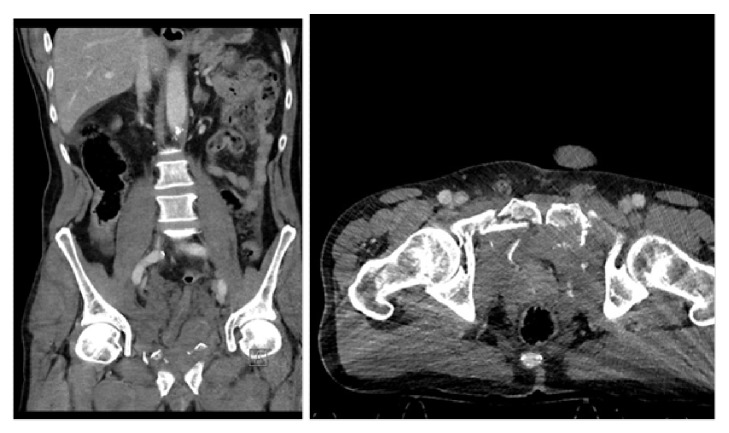
Contrast-enhanced CT: pelvic fracture and pelvic hematoma. The bladder cannot be assessed.

**Figure 2:**
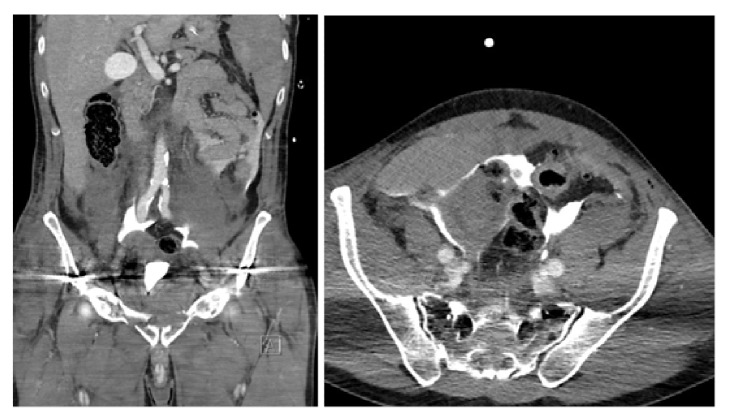
Bladder displaced upwards by the pelvic hematoma. Poor bladder filling with contrast extravasation in the peritoneal cavity.

**Figure 3:**
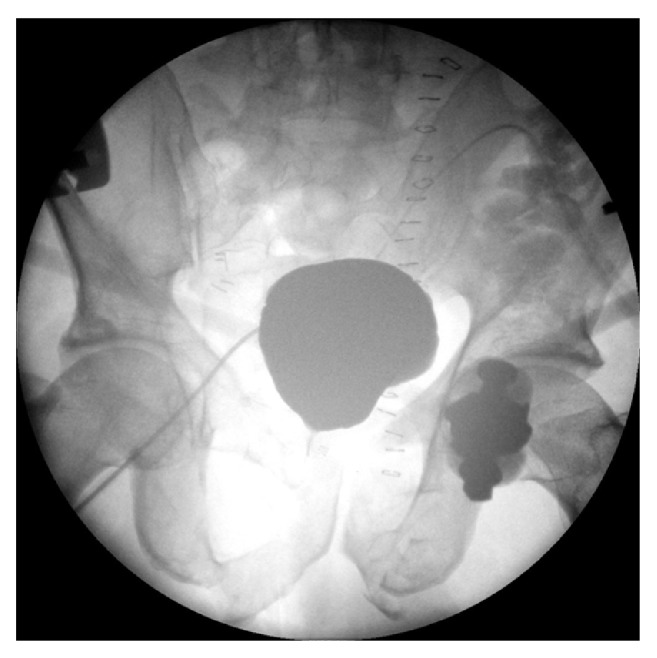
Cystography 10 days after surgical exploration and repair of the bladder injury. Note: the bladder is located high in the pelvis.

**Figure 4:**
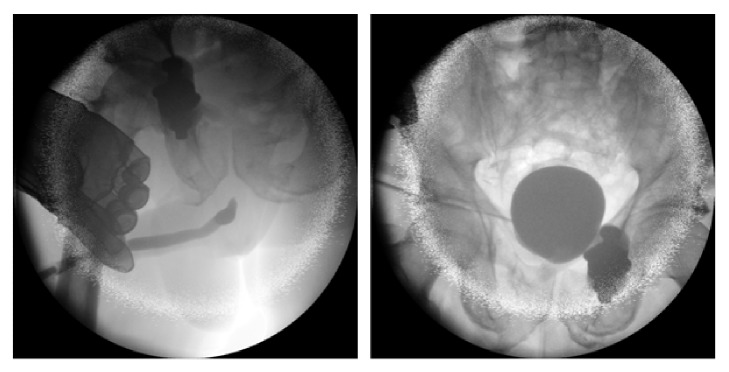
Combined retrograde urethrography (left) and antegrade cystourethrography 3 months after trauma and before urethroplasty.

**Figure 5:**
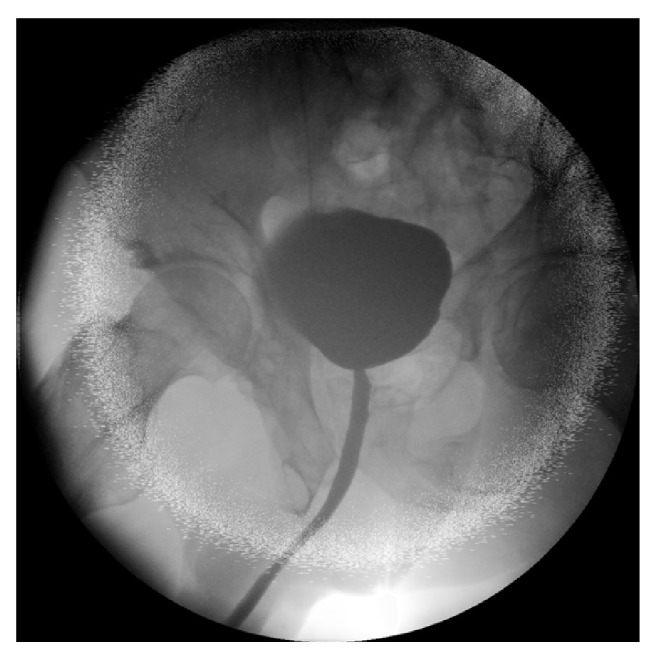
Voiding cystourethrogram 2 weeks after anastomotic repair urethroplasty.

A 59-year-old male was admitted to the nearest hospital after a serious car accident. The patient complained of pelvic and abdominal pain and had signs of hemodynamic instability (hypotension and tachycardia). Voiding was not possible. Clinical examination revealed pelvic instability and blood loss per urethra. Hemodynamic resuscitation was started and an urgent contrast enhanced CT-scan was performed. An excretory phase was not performed due to the patient's clinical condition. The CT-scan revealed a small liver laceration and an unstable pelvic fracture. The kidneys were normal and the bladder was empty (Figure 1). Immediate external fixation of the pelvis was performed. Postoperatively, the patient was transferred to the intensive care unit (4h after admission) for further resuscitation and monitoring. Urethral catheterisation was attempted but failed generating the 1st referral to urology. A suprapubic catheter was considered but the bladder could not be identified on abdominal ultrasound. The urologist decided to wait for adequate bladder filling and came back after 4 hours. The bladder was again not seen on ultrasound but free intraperitoneal fluid was demonstrated. A follow-up abdominopelvic CT-scan with excretory phase (14h after admission) demonstrated a nearly empty and upwards displaced bladder, with contrast extravasation into the peritoneal cavity (Figure 2). The urologist decided to place an intraperitoneal drain (percutaneously). On days 2 and 3 after the trauma, a progressive clinical deterioration evolved with signs and symptoms compatible with sepsis. Therefore, the decision was taken to transfer the patient to an academic hospital with dedicated trauma facilities. On admission, the patient was disoriented with hypotension, tachycardia, tachypnea, and abdominal pain. Clinical examination revealed marked abdominal distension and guarding. Laboratory blood tests demonstrated a severe electrolyte imbalance, an elevated creatinine level, and anaemia (Table 1). After multidisciplinary trauma-team discussion, immediate hemofiltration was arranged, followed by laparotomy. Copious amounts of urine were drained, and a large laceration in the bladder dome extending towards the extraperitoneal anterior surface of the bladder wall was observed. Direct inspection, via the laceration, of the ureteric orifices and bladder neck revealed no further injury. A suprapubic catheter was inserted and the bladder was closed in 2 layers (mucosa, detrusor) with a running suture monocryl™ 3.0. An extraperitoneal drain was placed and the intraperitoneal drain was maintained. Despite clinical indication of concomitant urethral injury (combination urethral blood loss-pelvic fracture, displacement of the bladder, and failed urethral catheterization), a trial of urethral realignment was not performed because of the clinical condition of the patient. During the following days, a clear clinical and biochemical improvement was noticed (Table 1). Ten days after laparotomy, a cystography proved integrity of the bladder wall repair (Figure 3). A flexible cystoscopy then revealed a complete urethral injury. Suprapubic drainage was maintained and combined retrograde and antegrade cystourethrography performed at 3 months showed a complete obliteration of the membranous urethra (Figure 4). The bladder had descended in the pelvis and the patient could lie in the lithotomy position. Urethroplasty (elaborated perineal approach with excision of scar tissue and anastomotic repair) was performed. The postoperative course was uneventful, voiding cystourethrogram (2 weeks after urethroplasty) proved no urethral extravasation and the catheter was removed (Figure 5). At present (27 months after the initial trauma), the patient is continent without voiding difficulties.

## 3. Case 2

**Figure 6:**
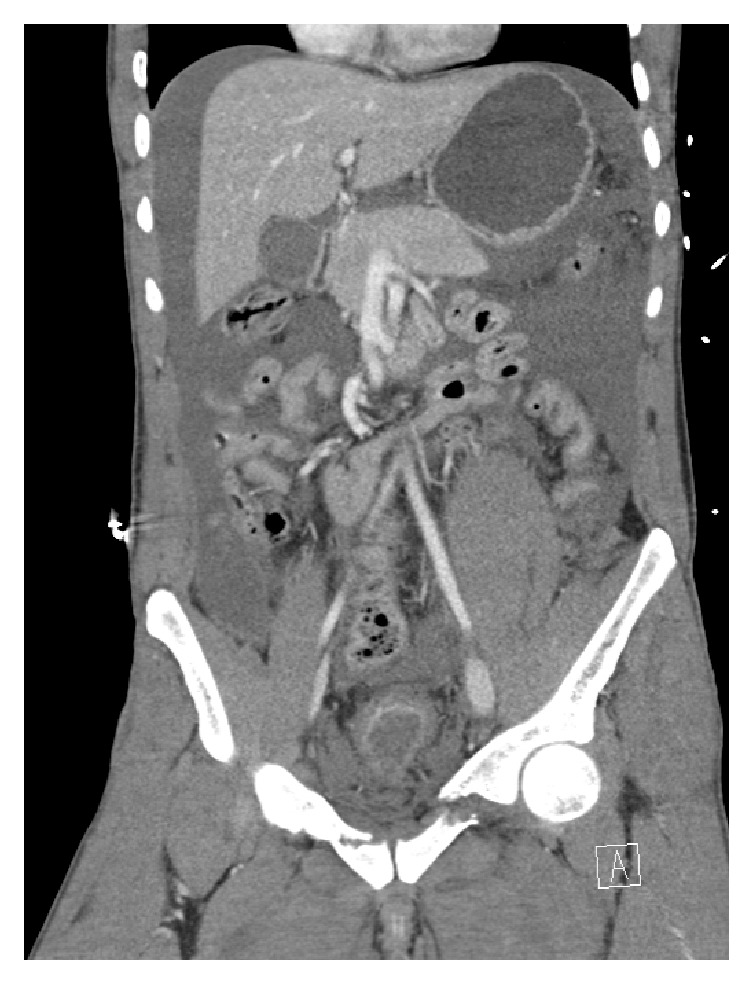
Unstable pelvic fracture with pelvic hematoma. Intraperitoneal free fluid.

**Figure 7:**
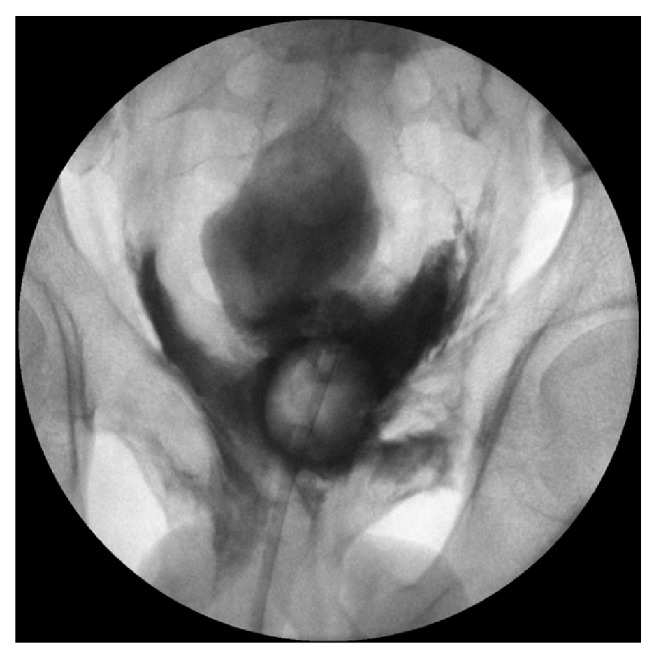
Conventional cystography: massive extraperitoneal extravasation at the bladder neck.

**Figure 8:**
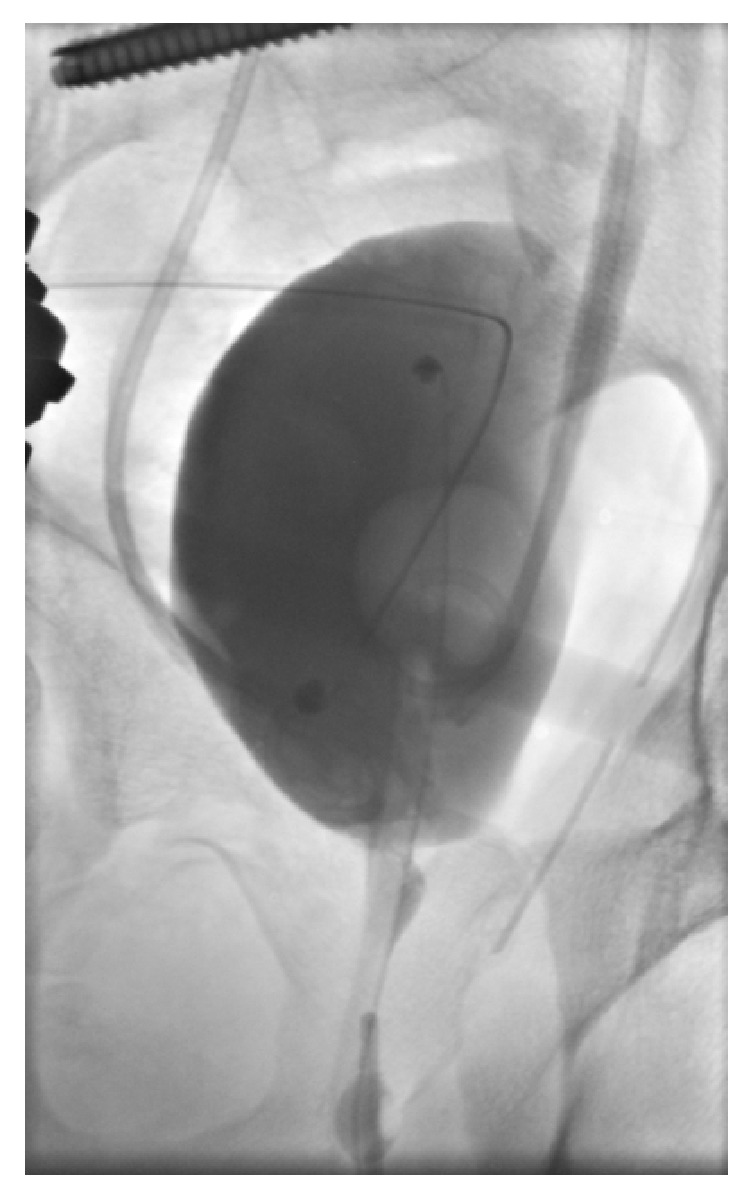
Postoperative cystography: full bladder wall integrity.

A 30-year-old male patient was run over by a lorry and immediately transferred to a dedicated trauma centre. Hemodynamic resuscitation was started and urgent contrast-enhanced CT was performed which revealed an unstable pelvic fracture together with large amounts of free fluid in the peritoneal cavity (Figure 6). A transurethral catheter was passed without difficulty but blood-stained urine was noted after insertion. A conventional cystogram demonstrated massive extraperitoneal extravasation at the bladder neck (Figure 7). Intraperitoneal extravasation was not noticed. The patient underwent immediate laparotomy and a large amount of urine was evacuated from the peritoneal cavity. Exploration revealed a large combined intraperitoneal and extraperitoneal tear of the bladder wall extending towards the bladder neck. The urethral catheter balloon was actually lying free in the pelvis outside of the bladder. The ureteric orifices were inspected and bilateral double-J stents were inserted. The bladder neck was repaired and the tear was closed with a 2-layer vesicorraphy. The urethral catheter was maintained, but additionally, a suprapubic catheter was inserted in the bladder. An abdominal and pelvic drain were left in place and the abdomen was closed. The postoperative course was uneventful and after 16 days, a pericatheter voiding cystourethrography demonstrated complete healing of the bladder wall (Figure 8). However, minimal extravasation was seen at the midbulbar urethra. At 6 weeks, the transurethral catheter was removed followed by cystourethroscopy which confirmed healing of urethra, bladder neck, and bladder wall. The double-J stents were removed as well. Spontaneous voiding was then possible without substantial residual volume of urine, and the suprapubic catheter was removed 3 days later. Patient, now 13 months after trauma, is voiding without any problems and is fully continent.

## 4. Discussion

After his car accident, the 1^st^ patient was admitted to the nearest hospital. Although this is common practice in most European countries, reduction of mortality (25%) and length of hospital stay (reflecting complication rate) has been reported if the patient is immediately transferred to a major trauma centre compared to a local hospital, even if the distance is greater (as in the 2^nd^ case)[4]. Although the EAU guidelines formulate a strong recommendation for transfer to a designated major trauma center, this is not always followed because of contemporary common practice in many regions of transferring the patient to the nearest hospital.

**(i) KEY MESSAGE 1: “Manage polytrauma patients in designated major trauma centres within a trauma network” (EAU strong recommendation)**

In unstable patients, immediate fluid resuscitation and diagnosis of life-threatening injuries are the priority. The standard diagnostic test is the contrast-enhanced CT-scan (“CT traumagram,” including head, neck, thorax, abdomen, and pelvis). From a urologic point of view, renal injuries will be diagnosed and staged by this traumagram [5]. In urgent situations, life-saving surgery cannot be postponed to await the excretory phase. In the 1^st^ case, the large bladder injury only became apparent at the 2^nd^ CT-scan with excretory phase. If contrast extravasation outside the bladder is noticed, the diagnosis of bladder injury is evident. However, passive bladder filling during the excretory phase is not sufficient to diagnose every bladder injury. The standard is cystography (plain or CT-cystography) with active bladder filling, as small bladder injuries might only become apparent after adequate (ca. 350ml) filling [6–8]. This requires a catheter in the bladder, which was not present during initial CT-scan in both cases. In the 2^nd^ case, intraperitoneal bladder injury could only be suspected by the presence of free fluid in the peritoneal cavity. After insertion of a transurethral catheter, plain cystography was performed demonstrating an extraperitoneal bladder injury. However, the intraperitoneal component was not visible as the catheter was not actually in the bladder. The combination of intra- and extraperitoneal bladder injury, as in these cases, is relatively rare (5-8%) [7]. Although the guidelines strongly recommend cystography to diagnose/rule out bladder injury, this is not always possible in urgent situations because of hemodynamic instability, because a catheter was not inserted in the bladder yet or because of difficulties in passing a catheter in the bladder.

**(ii) KEY MESSAGE 2: “Perform cystography with active retrograde filling of the bladder with dilute contrast” (EAU strong recommendation)**

In both cases, the combination of an unstable pelvic fracture and lower urinary tract bleeding, blood at the meatus or blood on catheter insertion, is indicative of lower urinary tract injury (bladder or urethra) until proven otherwise [6, 9]. Combined urethral and bladder injury is present in 4.1-15% of cases [6]. The urethral injury in isolation is not life-threatening and therefore has a lower priority in the management of the severely injured trauma patient. Retrograde urethrography is the standard diagnostic investigation in case of suspicion of urethral injury. However, in an unstable patient this should be delayed until the patient has been stabilized [6]. This was the case in both patients and not performing immediate retrograde urethrography was justified. The urethra was only evaluated after 2 weeks by flexible cystoscopy in the 1^st^ case. Flexible cystoscopy is indeed a valuable alternative to retrograde urethrography [6], especially when it is logistically simpler. In the 2^nd^ case, a partial bulbar (anterior) urethral injury was diagnosed during urethrography after 16 days. The EAU guidelines leave the choice between flexible cystoscopy and retrograde urethrography upon the discretion of the treating physician.

**(iii) KEY MESSAGE 3: “In an unstable patient, postpone urethral imaging until the patient has been stabilised”.**

**(iv) KEY MESSAGE 4: “evaluate urethral injuries with flexible cystoscopy and/or retrograde urethrography” (EAU strong recommendation)**

In case of urethral injury, urinary diversion by either urethral or suprapubic catheter has to be performed as soon as possible [6]. An attempt of urethral catheterization is allowed. This was successful in the 2^nd^ case and sufficient as treatment as blunt partial anterior urethral injuries heals without subsequent stricture formation in up to 68% of cases [10]. In the 1^st^ case where transurethral catheterisation failed, suprapubic catheter must be placed. This must be done with sonographic guidance as the bladder might be displaced by a pelvic hematoma [6]. Insertion of the suprapubic catheter in the 1^st^ case was not possible as the bladder was not full. This can be due to hemodynamic shock and waiting a couple of hours until adequate bladder filling is justified. However, the large bladder rupture hampered adequate bladder filling despite fluid resuscitation and stabilization of the patient. Once the diagnosis of the intraperitoneal (or combined intra-extraperitioneal) bladder injury is made, laparotomy and bladder repair are mandatory [6]. Persistent urine leakage in the peritoneal cavity will lead to peritonitis, sepsis, and electrolyte imbalances as demonstrated by the 1^st^ case. An intraperitoneal drain will not solve this problem and can only be regarded as temporary damage control measure. On the other hand, uncomplicated extraperitoneal injuries can be managed by bladder drainage only, in the absence of associated injuries requiring surgical intervention [11]. In the 2^nd^ case, the correct decision of immediate laparotomy was taken and the patient's recovery was uneventful. The presence of a bladder neck injury was another imperative factor to proceed with immediate exploration and repair because of the risk of urinary incontinence if left untreated. The EAU guidelines formulate a strong recommendation for surgical exploration and repair once an intraperitoneal bladder injury has been diagnosed and the 1^st^ case demonstrated a nearly fatal outcome after nonadherence to this recommendation.

**(v) KEY MESSAGE 5: “Manage intraperitoneal bladder injuries caused by blunt trauma by surgical exploration and repair” (EAU strong recommendation)**

After surgical repair of the bladder, bladder healing was assessed by cystography after 10 and 16 days. Although this control cystography is still advised after repair of complex injuries or in case of risk factors of wound healing, it can be omitted after repair of a simple injury in a healthy patient [12].

During laparotomy in the 1^st^ case, it was decided not to perform a trial of urethral realignment because prolonged anaesthesia was not advisable in his clinical condition. However, if the patient was stable, a trial of realignment would have been justified. This can prevent stricture formation in up to 51% of cases, and if a stricture occurs, treatment might be easier [13]. However, this optimistic cure rate is biased by the selection of minor (and probably partial) injuries towards endoscopic realignment whereas unstable patients with severe trauma (and a higher likelihood of complete injury) were allocated to suprapubic diversion only initially. Nevertheless, suprapubic diversion with delayed urethroplasty (≥3 months after trauma) is always a good solution for complete posterior urethra disruption [6]. Despite the fact that early realignment is an option to treat urethral injuries, the EAU guidelines continue to strongly recommend the practice of suprapubic diversion with deferred urethroplasty because of the good results with this deferred urethroplasty [1]. Ideally, a prospective randomized trial comparing early realignment versus suprapubic diversion and deferred urethroplasty would identify the best strategy, but it is unlikely that such a trial will be ever conducted.

**(vi) KEY MESSAGE 6: “manage complete posterior urethral disruption with suprapubic diversion and deferred urethroplasty” (EAU strong recommendation)**

Before delayed urethroplasty, a combination of retrograde urethrography with antegrade cystourethrography is advised to evaluate location and extent of the stricture, estimate bladder descent, and evaluate the competence of the bladder neck. Urethroplasty should be performed only if the bladder and prostate have descended sufficiently, the scar tissue has been stabilized, and the patient is able to lie in the lithotomy position. The standard technique is resection of fibrotic tissue and anastomotic repair by an elaborated perineal approach [14]. Two weeks after the urethroplasty, a voiding cystourethrogram is performed to rule out urinary extravasation, and the catheter is removed. In case of urinary extravasation, the catheter is maintained and reevaluation is performed after additional one week.

These cases demonstrate that complicated clinical situations might interfere with practical implementation of the guidelines and might delay diagnosis and treatment of lower urinary tract injuries. Nevertheless, adherence to the guidelines is crucial to prevent additional morbidity and mortality.

## Figures and Tables

**Table 1:** Laboratory values.

	**Preop (day 3)**	**DAY 4**	**DAY 5**	**Ref**
**Na (mmol/L)**	125	135	142	*135-144*
**K (mmol/L)**	6.4	5.1	4.2	*3.6-4.8*
**Cl (mmol/L)**	90	103	108	*98-106*
**Urea (mg/dL)**	120	71	50	*13-43*
**Creat (mg/dL)**	6.7	1.95	0.74	*0.72-1.17*
**eGFR (mL/min)**	8.2	36.5	>90	*>90*
**CRP (mg/L)**	233.8	148.5	105.4	*<5*
**Hb (g/dL)**	8.5	8.0	8.2	*12.9-17.3*
